# Chronic exposure to neonicotinoids increases neuronal vulnerability to mitochondrial dysfunction in the bumblebee (*Bombus terrestris*)

**DOI:** 10.1096/fj.14-267179

**Published:** 2015-01-29

**Authors:** Christopher Moffat, Joao Goncalves Pacheco, Sheila Sharp, Andrew J. Samson, Karen A. Bollan, Jeffrey Huang, Stephen T. Buckland, Christopher N. Connolly

**Affiliations:** *Medical Research Institute, University of Dundee, Dundee, United Kingdom; and ^†^Centre for Research into Ecological and Environmental Modelling, University of St. Andrews, St. Andrews, United Kingdom

**Keywords:** nicotinic acetylcholine receptors, neuronal culture

## Abstract

The global decline in the abundance and diversity of insect pollinators could result from habitat loss, disease, and pesticide exposure. The contribution of the neonicotinoid insecticides (*e.g.,* clothianidin and imidacloprid) to this decline is controversial, and key to understanding their risk is whether the astonishingly low levels found in the nectar and pollen of plants is sufficient to deliver neuroactive levels to their site of action: the bee brain. Here we show that bumblebees (*Bombus terrestris audax*) fed field levels [10 nM, 2.1 ppb (w/w)] of neonicotinoid accumulate between 4 and 10 nM in their brains within 3 days. Acute (minutes) exposure of cultured neurons to 10 nM clothianidin, but not imidacloprid, causes a nicotinic acetylcholine receptor-dependent rapid mitochondrial depolarization. However, a chronic (2 days) exposure to 1 nM imidacloprid leads to a receptor-dependent increased sensitivity to a normally innocuous level of acetylcholine, which now also causes rapid mitochondrial depolarization in neurons. Finally, colonies exposed to this level of imidacloprid show deficits in colony growth and nest condition compared with untreated colonies. These findings provide a mechanistic explanation for the poor navigation and foraging observed in neonicotinoid treated bumblebee colonies.—Moffat, C., Pacheco, J. G., Sharp, S., Samson, A. J., Bollan, K. A., Huang, J., Buckland, S. T., Connolly, C. N. Chronic exposure to neonicotinoids increases neuronal vulnerability to mitochondrial dysfunction in the bumblebee (*Bombus terrestris*).

Insects pollinate >70% of our crops, contributing an estimated U.S.$215 billion to the global economy each year (1). In addition to their contribution to crop yield, insect pollinators can also improve the quality of the harvest (2). Beyond this, insect pollination provides ecosystem services that underpin biodiversity. Because of their clear importance in food security, global economics, and ecosystem stability, there is worldwide concern over the decline in insect pollinators, including wild and managed bees.

The known risks to insect pollinators include interacting pressures from parasites, disease, habitat loss, poor nutrition, and exposure to pesticides (1). A direct threat to insect pollinators is the use of insecticides that target the insect nervous system and are the principal means to control insect pests of crops, livestock, and people (3). The neonicotinoids are the most commonly used insecticide; it is widely accepted that very low levels exist in nectar (1.9 ppb) and pollen (6.1 ppb) (4); and they have been detected (3.8–13.3 ppb) in dead/dying but not healthy bees (5). Exposure to these chemicals extends beyond the period of crop flowering as relevant levels (tens of parts per billion) persist in the soil (6) and in nearby dandelions (*Taraxacum officinale*, 1–6 ppb) (5). Moreover, honeybees store food within their hives to maintain the colony’s growth during poor weather and to sustain the colony over winter (7, 8).

Growing evidence indicates that sublethal levels of neonicotinoids may cause deficits in brain function (9), olfactory learning (10), navigation (11, 12), and colony development (13–15), therefore implicating their use in bee decline. However, others have failed to detect any deficits (16, 17). The target site of neonicotinoids is the nicotinic acetylcholine receptors (nAChRs) that, in insects, are found exclusively within the brain. However, despite our knowledge on exposure levels in the environment (4), we do not know if neonicotinoids reach the insect brain at a functional dose that is capable of perturbing neuronal function.

A second class of cholinergic insecticides is the cholinesterase inhibitors, the carbamates and organophosphates, which exert their effect by increasing acetylcholine to toxic levels. The organophosphate chlorpyrifos is used to treat a number of crops on which bumblebees forage, including grasslands, cranberries, top fruit, oilseed rape, and potatoes. In honeybee colonies, chlorpyrifos is detected commonly in wax (24.5 ppb), pollen (53.3 ppb), bees (3.4 ppb), and honey (46 ppb) (7, 8). Assuming a dietary exposure of 46 ppb (w/w) in honey, this equates to 30.8 ppb (w/v) (88 nM). Recently, additive toxicity between the neonicotinoids and organophosphates has been reported at the cellular (9) and whole bee (10) level in honeybees. This study tracks the dietary intake of neonicotinoid into the bumblebee brain and assesses its impact on neuronal function and colony performance, alone and in combination with raised levels of acetylcholine.

The field data, as the original Excel file, are available from the Environmental Information Data Centre Hub (*http://eidchub.ceh.ac.uk/metadata*).

## MATERIALS AND METHODS

### ^3^H-Imidacloprid feeding

Sugar syrup (Koppert Biologic Systems, Berkel en Rodenrijs, The Netherlands) was laced with 10 nM imidacloprid containing a ^3^H-imdacloprid (specific activity = 40 Ci/mmol) radioactive tracer (American Radiolabeled Chemicals, St. Louis, MO, USA). The syrup was mixed by inversion overnight at room temperature in the dark. *Bombus terrestris* microcolonies of 20 intermediate sized bees (250–350 mg) sourced from 3 different colonies were fed syrup with or without imdacloprid tracer for 3 days. Microcolonies were maintained on a 12 hour light/dark cycle at room temperature. After 3 days, bee brains were removed by dissection and placed in scintillation cocktail, and each bee brain was counted individually.

### Stable isotope dilution liquid chromotography-mass spectrometry/mass spectrometry analysis of imidacloprid in brains of bees

Bees were fed with sugar syrup containing 10 nM imidacloprid for 3 days. Bee brains were dissected and frozen at −80°C prior to analysis. A total of 63–100 bee brains were pooled together for analysis (*n* = 3). To each sample, 1 ml *d*_4_-imidacloprid (10 ng/ml) in acetonitrile was added and dissociated on ice manually with a tissue homogenizer. The samples were then sonicated on ice (2 × 10 s) with an ultrasonicator probe. The homogenates were centrifuged at 13,000 rpm for 10 minutes, and the supernatant was dried in a vacuum dryer. The samples were then reconstituted in 50 *µ*l acetonitrile followed by addition of 950 *µ*l 0.1% formic acid in water. A solid phase extraction using Waters Sep-Pak C18 columns primed with 1 ml acetonitrile and preconditioned with 0.1% formic acid in 5% acetonitrile was used to enrich imidacloprid.

Liquid chromotography–mass spectrometry/mass spectrometry (LC-MS/MS) analysis was carried out using a Dionex 3000 LC system (Thermo Scientific, Hemel Hempstead, United Kingdom) linked to a Quantum Ultra Mass Spectrometer (Thermo Scientific) with an IonMax ESI interface. A C18 column (Pursuit, 3 *µ*m, 50 × 1 mm; Thermo Fisher Scientific, Waltham, MA, USA) with a precolumn (Pursuit 3, MetaGuard; Thermo Fisher Scientific) was used to separate analytes. Five microliters of sample was injected, and each sample was analyzed in duplicate.

The LC was operated under gradient conditions with mobile phases of water/formic acid (99.9:0.1) (A) and acetonitrile/formic acid (99.9:0.1) (B) at a flow rate of 0.1 ml/min at 30°C. The initial mobile phase composition was 95% A, which was held for 1 minute, followed by a linear gradient over 5 minutes to 95% B, held at 95% B for 1 minute, and then returned to 95% A over 1 minute. The analytical column was then equilibrated at the initial conditions for 2 minutes for a total run time of 10 minutes.

Detection was in a multiple reaction mode, with transitions for imidacloprid being 256–209 and 256–175 and *d*_4_-imidacloprid being 260.00–213.00. At the MS source, the voltage was set at 4500 V, sheath gas pressure at 50, ion sweep gas pressure at 5, auxiliary gas pressure at 0, and capillary temperature at 300°C. The tube length offset was set at 81, and collision energy at 18 V for both imidacloprid (256–209) and *d*_4_-imidacloprid (260–213) and at 20 V for imidacloprid (256–175). The scan width was 0.05 (m/z), and the resolution for Q1 and Q3 was 0.7 (full width at half maximum). The argon pressure at Q2 was 1.5 mTorr. The optimized tuned condition was achieved by an infusion of imidacloprid (at 5 *μ*l/min) to LC (0.1 ml/min, 80% B) using a T connector.

Data analysis was performed using XCalibur (version 2.0; Thermo Scientific) and LCQuan (version 2.5.6; Thermo Scientific). The extracted data were output to Microsoft Excel for further calculation.

### *B. terrestris* primary neuronal culture

*B. terrestris* neuronal cultures were generated from the mushroom bodies of late-stage pupae. Mushroom bodies were dissected in cold supplemented Leibovitz’s L-15 medium (22.2 mM glucose, 13.8 mM fructose, 128.5 mM sucrose, and 28.6 mM proline; Sigma-Aldrich, Paisley, United Kingdom) and pooled into ice-cold divalent cation-free Ringer solution (135 mM NaCl, 5 mM KCl, 180 mM sucrose, and 20 mM 4-(2-hydroxyethyl)-1-piperazineethanesulfonic acid, pH 7.2). Cells were trypsinized for 6 minutes and then incubated in 1 mg/ml trypsin inhibitor for 5 minutes. Cells were centrifuged for 1 min, 500 rpm, at room temperature. Supernatant was removed, and cells were resuspended and titurated in warm (28°C) supplemented L-15 medium. After being allowed to settle for 2 minutes, cells were plated onto poly-d-lysine (1 mg/ml)–coated glass coverslips. Cultures were maintained in the dark at 28°C in supplemented L-15 medium.

### LIVE/DEAD viability/cytotoxicity assay

Viability assays on *B. terrestris* primary neuronal cultures were carried out using the LIVE/DEAD viability/cytotoxicity kit (Invitrogen, Carlsbad, CA, USA). Cells were pretreated with pesticides for 24 hours and then washed with phenol red free-supplemented L-15 medium. Cells were stained for 30 minutes in the dark at room temperature with a dye cocktail (4 *µ*M EthD-1, 2 *µ*M Calcein AM) made up in phenol red free-supplemented L-15 medium. Cells were washed for 5 minutes, imaged using an inverted wide-field imaging system, and analyzed using Volocity (PerkinElmer, Waltham, MA, USA) software. Excitation/emission (Ex/Em) wavelengths and bandwidth (in square brackets) used for the fluorescent dyes were Calcein AM (Ex/Em = 492[18]/535[30]) and EthD-1 (Ex/Em = 572[23]/630[60]). Multiple fields of view were imaged from each coverslip.

### JC-1 detection of mitochondrial membrane potential

*B. terrestris* primary neuronal cultures were washed with phenol red-free–supplemented L-15 medium and then incubated in the dark at 28°C for 15 minutes in 1 *µ*g/ml JC-1 (5,5′,6,6′ -tetrachloro-1,1′,3,3′-tetraethylbenzimidazolcarbosyanine iodide; Invitrogen) made up in phenol red-free–supplemented L-15 medium. Cells were then washed with phenol red-free–supplemented L-15 medium for 15 minutes in the dark at 28°C. Cells were imaged live in phenol red-free–supplemented L-15 medium using an inverted wide-field imaging system and analyzed using Volocity (PerkinElmer) software, and chemical additions were added as 2× stock (300 *µ*l). Images were obtained under ×400 magnification using excitation/emission wavelengths and bandwidth (in square brackets) as follows: for polarized mitochondria (red; Ex/Em = 572[23]/630[60]) and depolarized mitochondria (green; Ex/Em = 492[18]/535[30]) with a 30 second capture rate.


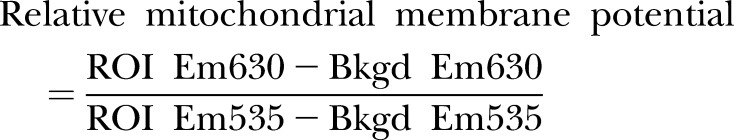


### Acetylcholinesterase assay

Bumblebee brains were extracted by dissection and homogenized in PBS. Protein concentrations were determined by the Bradford assay, and acetylcholinesterase (AChE) activity was assayed at 14 mg/ml. AChE activity was determined using the Ellman assay. AChE inhibitors (appropriate concentrations) were incubated in bumblebee brain lysates for 20 minutes. Samples were then incubated at room temperature with a reaction mix containing the color indicator 50, 50 dithiobis (2-nitrobenzoic acid) (286 mM), and acetylcholine (ACh) iodide substrate (0.86 mM) for 30 minutes, and AChE activity was monitored by absorbance at 412 nm. AChE activity was normalized to control measurements. IC_50_ values were obtained from Hill equation fits of the data from 3 independent experiments.

### Field experiment

Bumblebees (*B. terrestris audax*, the buff-tailed bumblebee) were housed 3 nests to a box, with entrances at the 2 ends and 1 in the middle. Two Tripols were assigned to each treatment group and placed in the field with ∼1 m spacing between each Tripol. Therefore, any orientation mistakes (14) would be contained within a treatment group. The colonies were sited in a sheltered position within a wilderness/enriched grassland habitat in Wester Ross, The Highlands, Scotland. In this area, total pesticide (arable and grassland use) load is much reduced (∼130-fold), as is the use of insecticides (∼5000-fold) compared with intensively farmed arable areas such as East Fife (Scotland) (Supplemental Table S1, data provided by Science and Advice for Scottish Agriculture). No neonicotinoid or organophosphate use was encountered on farms sampled in the Highlands and Islands indicating that environmental contamination with these compounds is unlikely.

Treatment was provided in the form of pesticide addition to the supplemental sugar syrup feed provided with colonies. All colonies were provided with 1500 ml of sugar syrup containing the appropriate pesticide or were left untreated. Once spiked, colonies were closed and transported to the field site where they were opened within a day of exposure to treatment. At this point, bees were free flying throughout and were not forced to consume the sugar syrup provided. No pollen was provided and bees needed to forage for this. The order of the treatment boxes at the site was UT, single treatment, and double treatment to minimize any local effects. Experiments were performed on 2 separate occasions, with the first comparing untreated, chlorpyrifos (150 nM) and chlorpyrifos (150 nM)/imidacloprid (10 nM). The second experiment was placed on the same site and consisted of untreated, imidacloprid (10 nM)/chlorpyrifos (150 nM) plus imidacloprid (10 nM). Colonies were place in the field for 43 (second experiment; June 28–August 9, 2014) or 48 (first experiment; April 25–June 11, 2014) days (as access to the site permitted).

On the final day of the trial, entrance gates were set to permit bee entries only (no exits) and after ≥5 hours (the average foraging duration for bees exposed to imidacloprid is 42 minutes), the entrance gates were closed, and colonies returned to the laboratory for assessment. Colony assessment was determined by increase in colony mass, total live number of bees remaining, average bee mass, the number of healthy brood cells on the surface of the nest, and overall condition of the nest (Supplemental Fig. S2). Each individual nest mass was recorded at the beginning and end of the experiment (excluding the sugar syrup feed provided). Colonies were then anesthetized with CO_2_, and live bees (identified by a combination of appearance and movement when handled) were removed, weighed, and euthanized quickly in ice-cold water containing detergent so that they didn’t awaken.

### Statistical analysis

We pool the data from the 2 field experiments. In our models, nests are nested within boxes, which allow us to incorporate any box effects and absorb any experiment effect into the box effects.

We used the following generalized linear mixed models:

1. Number of live bees/number of brood cells: A quasipoisson model with log link function was assumed. C and I were included as main effects (thus C = I = 0 corresponds to the control, C = 1 I = 0 to chlorpyrifos alone, C = 0 I = 1 to imidacloprid alone, and C = I = 1 to both chlorpyrifos and imidacloprid). Box was also included as a main effect, with nests nested within boxes.
2. Mean mass of live bees in nest/total bee mass in nest: A *γ* model with log link function was assumed. C and I were included as main effects. Box was also included as a main effect, with nests nested within boxes.
3. Final nest mass. Model same as for mean mass, except that log(initial nest mass) was included as a covariate, to adjust for any variation in initial nest size. For each model, we also tested for evidence of an interaction between C and I.

## RESULTS

**Figure 1.**
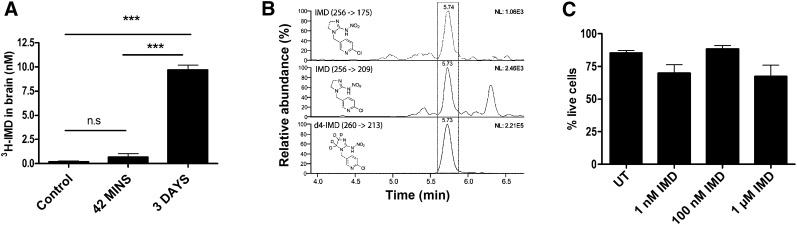
Imidacloprid accumulation in the brain does not affect neuronal viability. *A*) Bumblebees were fed radioactive imidacloprid (^3^H-IMD) for times indicated, and brains (10 bees, *n* = 3) were isolated and counted by scintillation to determine imidacloprid concentration. ***P < 0.001 (1-way ANOVA with Bonferroni’s multiple comparison test). *B*) Bumblebees were fed imidacloprid for 3 days, and the brain was excised and analyzed by stable isotope dilution LC/MS to determine the concentration of active ingredient [imidacloprid (transition 256→175 and 256→209)] with an internal standard [*d*_4_-imidaclorid (260→213, 500 pg on-column)]. Examples of ion chromatograms from a bumblebee brain extract are shown. *C*) Bumblebee brain neurons (DIV 3–10) were exposed to imidacloprid (1 nM to 1 *μ*M) for 24 hours, and cell viability was determined using calcein AM/EthD-1 staining (∼8–11 fields, ∼50 neurons per field, *n* = 3).

To determine the delivery of neonicotinoid to the brain following dietary intake of field relevant levels, adult bumblebees (*B. terrestris audax*) were fed sugar syrup containing imidacloprid [10 nM, 2.1 ppb (w/w)]. For rapid and sensitive detection, we tracked the accumulation of ^3^H-imidacloprid. To exclude external contamination of the head and proboscis, we excised the brains for analysis and determined the concentration on the basis of the average size of a bumblebee brain (1.16 *μ*l) (18). We find that imidacloprid (or its metabolites) does not reach significant levels within 42 minutes (an average foraging flight for bees exposed to imidacloprid) (14) but does accumulate to 9.7 ± 0.8 nM after 3 d (**Fig. 1*A***). The presence of intact (nonmetabolized) imidacloprid (at 3 days) was confirmed by using stable isotope dilution LC-MS (Fig. 1*B*) to be between 4.2 ± 1.7 (transition 256–209) and 5.2 ± 1.7 nM (256–175) (Supplemental Fig. S1). Imidacloprid is not lethal to brain neurons in culture (1 *μ*M, 24 hours; Fig. 1*C*) or fed caged bees (10 nM, 5 days; data not shown). Therefore, any toxicity to adult bees is likely limited to neuronal dysfunction rather than acute brain damage.

**Figure 2.**
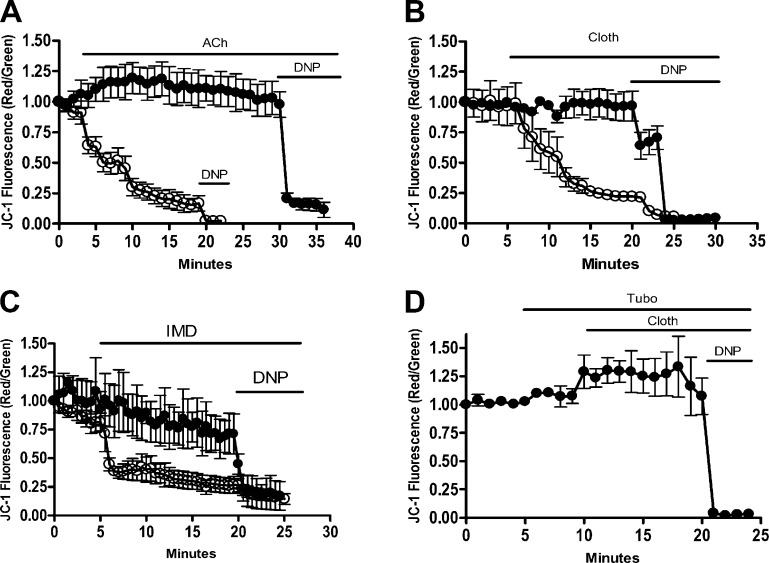
Bumblebee brain neurons undergo mitochondrial depolarization when nAChR are hyperstimulated. *A*) Bumblebee neurons in culture undergo mitochondrial depolarization in the presence of high levels (1 mM; open circles) but not low levels (100 *μ*M; filled circles) of acetylcholine. The neonicotinoid, clothianidin (*B*), induces mitochondrial depolarization at 10 nM (open circles) but not at 1 nM (filled circles), and imidacloprid (*C*) induces mitochondrial depolarization at 1 *µ*M (open circles) but not at 10 nM (filled circles). (D) Neurons pre-exposed to the nAChR antagonist *d*-tubucurarine (500 *μ*M) do not undergo mitochondrial depolarization in the presence of clothianidin (100 nM), demonstrating an nAChR-dependent process. In all cases, mitochondrial depolarization was monitored using ratiometric (red/green) JC-1 imaging, and the experiment was terminated by full mitochondrial depolarization using 2,4-dinitophenol (1 mM). In all cases, 15–20 regions of interest were monitored (*n* = 3).

As neurons are energetically demanding cells that require mitochondrial ATP production to maintain ion homeostasis (19), a constant mitochondrial membrane potential is critical for normal neuronal function (20). In mammalian neurons, excessive excitatory stimulation (by glutamate or its synthetic agonists) causes mitochondrial dysfunction (19) and long-term neural deficits (20). Therefore, we investigated whether the insect excitatory neurotransmitter, ACh, or its synthetic neonicotinoid agonists could influence mitochondrial function in bumblebee neurons. We find that exposure to high levels of ACh (1 mM, but not 100 *μ*M), induces acute mitochondrial depolarization (**Fig. 2*A***). In contrast, both clothianidin (Fig. 2*B*) and imidacloprid (Fig. 2*C*) can induce acute mitochondrial depolarization at much lower levels (10 nM and 1 *µ*M, respectively). As in mammals (19), this effect is receptor dependent and is blocked by the nAChR antagonist tubocurarine (500 *μ*M; Fig. 2*D*). Therefore, on the basis of the accumulation of imidacloprid in bumblebee brains, an exclusive dietary exposure (over days) to clothianidin is sufficient to cause acute brain mitochondrial dysfunction in bumblebees. In contrast, for imidacloprid, the dose reached (5–10 nM) is insufficient to induce mitochondrial depolarization when presented acutely (30 minutes). However, the risk to bumblebees results from chronic exposure over many weeks during crop flowering and perhaps even longer due to its persistence in the soil (6) and re-emergence in wildflowers (5).

**Figure 3.**
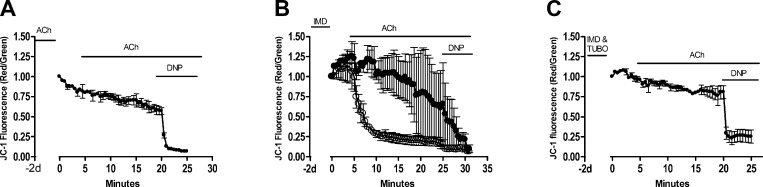
Chronic exposure to low levels of imidacloprid increases mitochondrial vulnerability. Bumblebee neurons (3–10 DIV) exposed chronically for 2 days (−2 days to 0 minutes) to (*A*) ACh (100 *μ*M) do not induce vulnerability to mitochondrial depolarization by a subsequent exposure to subeffect ACh (100 *μ*M). *B*) Low level imidacloprid (1 nM) induces vulnerability to a subsequent exposure to subeffect ACh (100 *μ*M), revealing fast responders (open circles) and slow responders (filled circles). Nonresponders not shown. *C*) Low level imdacloprid (1 nM) and tubocurarine (500 *µ*M) coexposure prevents development of mitochondrial vulnerability to subeffect ACh (100 *µ*M). In all cases, mitochondrial depolarization was monitored using JC-1 and the experiment was terminated by full mitochondrial depolarization by 2,4-dinitophenol (1 mM).

Even if the length of exposure may be extended, in a real landscape, alternative forage may be available, and therefore the actual exposure level may be reduced. Therefore, we probed further for potential deficits at even lower concentrations and over a longer duration. Neurons exposed chronically to ACh (100 *µ*M, 48 hours) do not become sensitized to ACh, and they are resistant to a subsequent acute exposure to ACh (100 *μ*M; **Fig. 3*A***). In contrast, although low-level imidacloprid (10 nM) does not induce mitochondrial depolarization acutely (Fig. 2*C*), when neurons are exposed chronically (48 hours) to just 1 nM imidacloprid, vulnerability to the normally innocuous ACh (100 *μ*M) exposure occurs (Fig. 3*B*). Under these conditions, mitochondrial responses to imidacloprid can be divided into 3 cell groups; nonresponders (49.6 ± 21.2%; data not shown) and neurons undergoing mitochondrial depolarization either rapidly (37.4 ± 31.0%) or slowly (13.0 ± 11.4%). To confirm that the development of vulnerability to mitochondrial depolarization is receptor dependent, as seen for the acute effects of clothianidin (Fig. 2*D*), tubocurarine (500 *μ*M) was included during the chronic exposure period (48 hours) to 1 nM imidacloprid. Under these conditions, no increased vulnerability to ACh (100 *μ*M) occurs (Fig. 3*C*), confirming that sustained nAChR activation is required to establish mitochondrial vulnerability to ACh.

Given the impact of neonicotinoids shown here, bee brain neurons would be unable to generate the energy required for homeostatic control and neuronal function. The accumulated loss of adult bee performance and/or developmental consequences to the brood, as seen previously at higher doses (20, 21), could impact colony growth (13, 14). Therefore, we replicated our feeding regime on whole bumblebee colonies to relate our cellular responses to colony performance. Bees were allowed to forage freely throughout the experiment in a predominantly wilderness environment, where few pesticides and no neonicotinoids or organophosphates, are used (Supplemental Table S1). As neonicotinoids increase the vulnerability of bee neurons to ACh, we were determined to increase ACh levels by coexposure to a cholinesterase inhibitor. We determined the IC_50_ (4.47 ± 0.16 nM) for the chlorpyrifos oxon active metabolite (of chlorpyrifos) in bumblebee brains to be well below the likely environmental dose (∼88 nM) (7, 8).

Therefore, bumblebee colonies were provided with field relevant levels of imidacloprid (10 nM) and/or chlorpyrifos (150 nM) in sugar syrup and left at a single site to forage freely for 43–48 days. Three nests (all treated identically) were housed in each box. The individual values of all nests are indicated for colony growth, number of live bees and viable brood, and the individual bee masses plotted.

**Figure 4.**
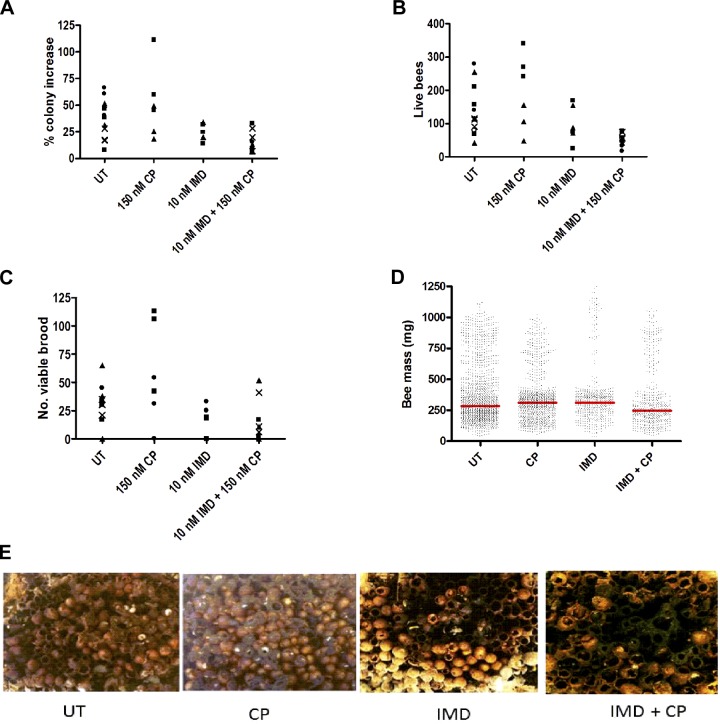
Exposure of bumblebee colonies to field-relevant levels of imidacloprid decreases colony performance. Bumblebee colonies of similar mass were provided with sugar syrup (UT, *n* = 12), containing chlorpyrifos (CP, 150 nM, *n* = 6), imidacloprid (IMD, 10 nM, *n* = 6), or both (IMD + CP, *n* = 12). No pollen was provided, and bees were free to forage in a wilderness/grassland area in the west of Scotland. After 43–48 d in the field, colony performance indicators were monitored. Nests within each box are depicted with the same symbol. *A*) Percentage colony mass increase for each nest. *B*) Total number of live bees remaining in each nest. *C*) Number of viable brood cells on the outer face of each nest. *D*) Size distribution scatter of individual bee masses within each treatment group. Median values are shown by red lines. *E*) To report on the condition of the nests, a representative image of each nest was collected. A representative example from each treatment group is illustrated (all images are available in Supplemental Fig. S2).

As expected for a natural environment, colony performance was variable, even in untreated colonies. Colony growth was significantly impaired in colonies exposed to imidacloprid (imidacloprid, 24.0 ± 1.0% or imidacloprid + chlorpyrifos, 14.4 ± 3.6%) compared with untreated colonies (38.0 ± 15.3%), or chlorpyrifos alone (51.5 ± 29%) (**Fig. 4*A***, individual nest values indicated). Similarly, the number of surviving bees was reduced significantly in the presence of imidacloprid (imidacloprid alone, 97.2 ± 11.1; imidacloprid/chlorpyrifos, 53.3 ± 14.1) compared with untreated (138.7 ± 24.7) or chlorpyrifos-treated (193.5 ± 127.5) colonies (Fig. 4*B*, individual nest values indicated). Finally, to indicate future colony potential, viable brood cell number on the exterior face of the nest was determined. Again, compared with untreated (32.7 ± 6.7) and chlorpyrifos-treated colonies (57.7 ± 41.5), this was reduced significantly by imidacloprid (imidacloprid alone, 15.8 ± 5.0; imidacloprid/chlorpyrifos, 12.0 ± 9.6) (Fig. 4*C*, individual nest values indicated). In all cases, there was no significant impact of chlorpyrifos on the deficits caused by imidacloprid. We observed no significant difference in the average bee mass for any treatment group (Fig. 4*D*). Finally, nest condition in the presence of imidacloprid was severely compromised by fungal contamination (Fig. 4*E*, see Supplemental Fig. S2 for images of all nests), and some weakened colonies were overrun by wasps (*Vespula vulgaris*).

Differences in colony performance were assessed statistically using generalized linear mixed models (**Tables 1** **and** **2**). The interaction between chlorpyrifos and imidacloprid was not significant at the 5% level for any of the analyses, and therefore we report results of fitting the models without interaction. In 2 cases, number of live bees (*P* = 0.057) and final nest mass (*P* = 0.085), the interaction was significant at the 10% level, providing weak evidence that the effect of imidacloprid was greater in the presence of chlorpyrifos. In Table 1, we show *P* values for testing the null hypotheses of no treatment effect on each response variable. These results show no indication of an effect of chlorpyrifos, whereas there was evidence of an effect of imidacloprid on all response variables except the mean mass of live bees (Table 1). For the other 4 response variables, the 95% confidence intervals for the coefficient of imidacloprid, together with the corresponding interval for percent reduction in the response variable in the presence of imdicaloprid, are shown (Table 2).

**TABLE 1.** Tests of the null hypothesis of no treatment effect for chlorpyrifos (C) and imidacloprid (I)

Response	C	I
Number of live bees	0.822	0.009
Number of healthy brood cells	0.314	0.006
Mean mass of live bees	0.426	0.978
Total bee mass in nest	0.395	0.028
Final mass of nest	0.906	0.012

Tabulated values are *P* values.

**TABLE 2.** Estimates and 95% confidence intervals for the coefficient of imidacloprid

Response	Estimated coefficient	95% confidence interval	Estimated % reduction	95% confidence interval
No. live bees	−0.81	(−1.36, −0.26)	55%	(23%, 74%)
No. healthy brood cells	−1.22	(−2.00, −0.45)	71%	(36%, 86%)
Total bee mass in nest	−0.85	(−1.58, −0.12)	57%	(11%, 79%)
Final mass of nest	−0.19	(−0.32, −0.05)	17%	(5%, 27%)

The coefficient would be zero in the absence of an effect; negative values indicate a negative impact of imidacloprid. Also shown are the corresponding estimates and 95% confidence intervals for the percent reduction of the response variable in the presence of imidacloprid.

## DISCUSSION

In terms of risk from neonicotinoids to bees, a prerequisite is that neonicotinoids reach a pharmacologically relevant level at their site of action: the insect brain. In this study, we demonstrate the delivery of neuroactive levels of imidacloprid to the brains of bumblebees fed at a field realistic level for 3 days. Brain levels were determined by both the use of a radioactive tracer and LC-MS to confirm, beyond doubt, the existence of active parental compound in the brain. This is likely an underestimate of exposure to active ingredient as the imidacloprid metabolite, olefin, is neuroactive in bees (9) and toxic to pests (22). Within the duration of a typical foraging bout (42 minutes when exposed to neonicotinoids) (14), no imidacloprid is detected. Therefore, no immediate impairment on bee function, such as homing ability, would be expected after initial exposure to normal levels of neonicotinoids. However, after approximately 3 days, imidacloprid accumulates to low nanomolar levels. Interestingly, even high levels (1 mM) of imidacloprid do not kill bumblebee neurons over 24 hours. Therefore, the consequences of normal neonicotinoid exposure would be expected to be subtle. Indeed, caged bees fed this level of imidacloprid over several days did not die (data not shown).

In terms of neuronal function, we observe that a low level of clothianidin (10 nM) activation of nAChRs does cause acute mitochondrial depolarization, making it 100,000-fold more potent, in this respect, than acetylcholine. Exposure to imidacloprid at this level (as realized after 3 days dietary exposure) did not cause acute (<25 minutes) mitochondrial depolarization. However, under the more realistic conditions identified in this study (present at <10 nM for days), as little as 1 nM imidacloprid increases neuronal sensitivity to acetylcholine, where a normally innocuous level (100 *μ*M) is now capable of inducing mitochondrial depolarization in the majority of neurons.

One possible mechanism of increased sensitivity to acetylcholine is a pharmacological chaperone type effect by neonicotinoids, leading to an up-regulation in nAChR expression. In support of such a hypothesis, changes in nAChR expression have been observed in mammals exposed chronically to nicotine (23, 24) and even neonicotinoids (25). Although high levels (19–70 *μ*M) of neonicotinoids were required to up-regulate mammalian receptors, this most likely reflects their low affinity for the mammalian receptors, and a similar up-regulation of insect nAChRs may occur at much lower, field-realistic, levels.

Mitochondrial dysfunction compromises ATP production and therefore disrupts neuronal homeostasis, plasticity, learning, and behavior in mammals (19, 26). Importantly, the neurons investigated here are Kenyon cells that constitute >40% of cells in the bee brain (27). They are the major neuronal component of the mushroom bodies, a higher-order insect brain structure that mediates multisensory integration, learning, and memory (28, 29). Therefore, mitochondrial dysfunction in Kenyon cells provides a reasonable explanation for the memory deficits (10) and poor navigation (11, 12) observed in honeybees and the reduced foraging efficiency in bumblebees (14, 30) exposed to neonicotinoids. Under more chronic conditions, a contribution from endogenous acetylcholine during intense synaptic activity, when bees are learning to forage on new flowers or in new areas, is likely. Therefore, the impact on colonies may be greater in challenging landscapes or weather conditions (31).

Previous colony studies used different conditions, with higher levels of imidacloprid (6), or included the exposure in both sugar and pollen (5), and colonies were laboratory based throughout (6) or during the period of exposure to imidacloprid (5). Therefore, to directly relate to our cellular studies, we performed a field trial on bumblebee colonies in a wilderness environment using the feeding regime that we used to track imidacloprid into the brain and assess its consequences. To mimic enhanced exposure to acetylcholine, we included a field-relevant level of the organophosphate chlorpyrifos in the sugar solution provided. Importantly, bees had to forage for their own pollen if they were to be successful at raising brood. Therefore, colonies suffering a deficit in their foraging ability (*e.g.,* olfactory learning or navigation) should fail to grow as strongly as control colonies. Chlorpyrifos, when present alone, exerted no significant effect on colony performance. In contrast, in colonies exposed to imidacloprid, few colonies exhibited strong nest growth, and they had fewer bees and brood cells. In the honeybee, very high doses (50–75 *µ*M) of imidacloprid directly act on mitochondrial function (32), whereas at very low doses in the diet (0.15 pM), mitochondrial structure is normal in the midgut after 8 d of feeding (33).

The failure of chlorpyrifos to enhance the effect of imidacloprid may reflect that the negative impact of imidacloprid is already maximal. Accumulating evidence suggests that neonicotinoids (at field-relevant levels) exert their toxicity by a chronic deficit in neuronal function (9), leading to deficits in learning and memory (10) and poor colony foraging capacity (14, 30). Therefore, the effect of neonicotinoids on insect colonies may depend on how challenging the environment is in terms of food availability and weather (foraging opportunities). In our field trial, the area is typically wet and windy, and there was little garden or commercial forage available, suggesting that small deficits in foraging efficiency, compounded over time, may have had a high impact on our colonies.

The consequences of neonicotinoid exposure may be exacerbated by the coexistence of other environmental threats such as disease (34), other pesticides (7), or exposure to other sources of neonicotinoids from nearby wildflowers (5) or treated lawns (35), as synergistic interactions between neonicotinoids have been reported (patent no. U.S. 7,745,375 B2; 2010). Our study indicates that the consequences of neonicotinoid exposure would be subtle, affecting higher cognitive function. This is consistent with previous studies identifying deficits in learning (10), navigation (11, 12), foraging (14, 30), and colony growth (13, 14). Importantly, such deficits would be delayed while the impact of decreased foraging performance accumulates within a colony, and this has been reported (13, 14, 36). On the basis of imidacloprid accumulation, this study indicates that an acutely effective dose of clothianidin or a chronically effective dose of imidacloprid reaches the bumblebee brain within 3 days of dietary exposure to neonicotinoids. Future field trials will need to consider whether bees are challenged sufficiently (in terms of pesticide exposure time, forage availability, weather, and disease) if cognitive deficits resulting from pesticide exposure are to be revealed. Indeed, the improvement of forage availability for all insect pollinators may help to mitigate the negative impact of insecticides.

## Supplementary Material

Supplemental Data

## Abbreviations

ACh: acetylcholine
AChE: acetylcholinesterase
Ex/Em: excitation/emission; JC-1, 5,5′,6,6′ -tetrachloro-1,1′,3,3′-tetraethylbenzimidazolcarbosyanine iodide
LC-MS/MS: liquid chromotography-mass spectrometry/mass spectrometry
nACh: nicotinic acetylcholine receptor
