# Supplementary material for: Chronic exposure to neonicotinoids increases neuronal vulnerability to mitochondrial dysfunction in the bumblebee (Bombus terrestris)
Source: FASEB J. 2015 Jan 29;29(5):2112–9. doi: 10.1096/fj.14-267179 (PMC4415021; doi:10.1096/fj.14-267179)
Supplement: Supplemental Data [file supp_29_5_2112__index.html]

Chronic exposure to neonicotinoids increases neuronal vulnerability to mitochondrial dysfunction in the bumblebee (Bombus terrestris) — Supplemental Data 

# Chronic exposure to neonicotinoids increases neuronal vulnerability to mitochondrial dysfunction in the bumblebee (*Bombus terrestris*)

## Supplemental Data

**Files in this Data Supplement:**

- Supplemental Data
- Supplemental Data
- Supplemental Data
