# Supplementary figures and images for: Chronic exposure to neonicotinoids increases neuronal vulnerability to mitochondrial dysfunction in the bumblebee (Bombus terrestris)
Source: FASEB J. 2015 Jan 29;29(5):2112–9. doi: 10.1096/fj.14-267179 (PMC4415021; doi:10.1096/fj.14-267179)

A

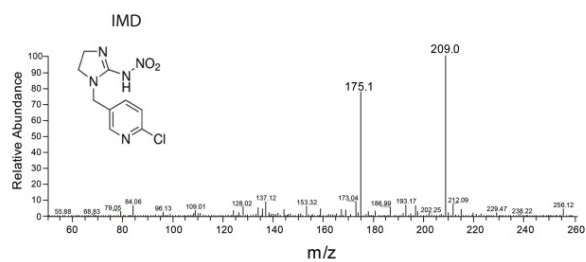

B

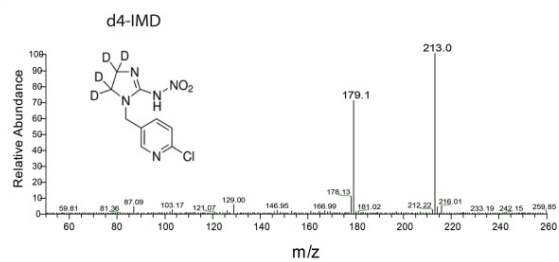

C

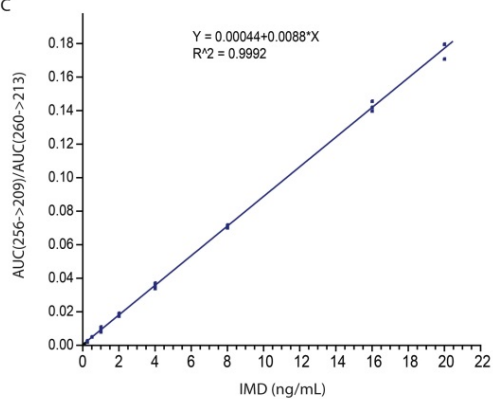

D

|                | N | [IMD] in bee brain<br>(assuming brain<br>volume=<br>1.16uL)(nM) |
|----------------|---|-----------------------------------------------------------------|
| IMD (256->209) | 3 | 4.2 ± 1.7                                                       |
| IMD (256->175) | 3 | 5.2 ± 1.7                                                       |

Supplement: Supplemental Data [file supp_fj.14-267179_Supplemental_Figure1.pdf]

25th APRIL 2014- 11th JUNE 2014

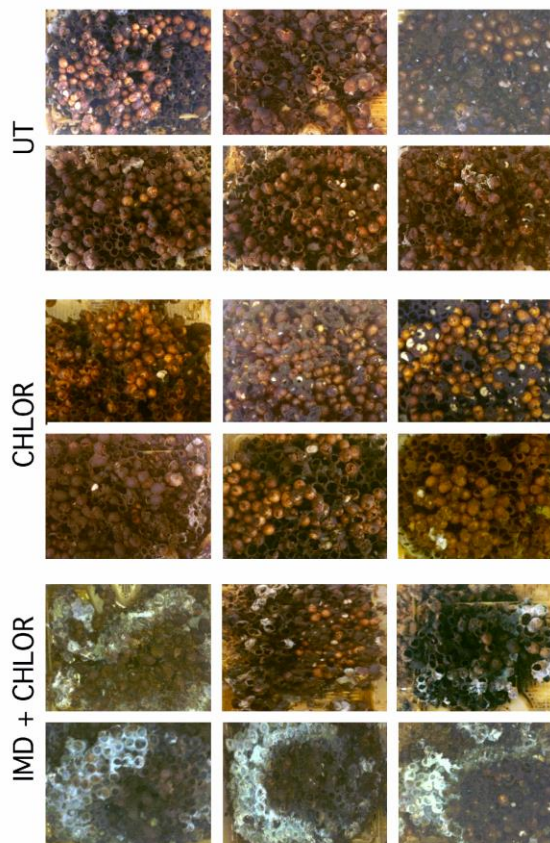

28th JUNE 2014 - 9th AUGUST 2014

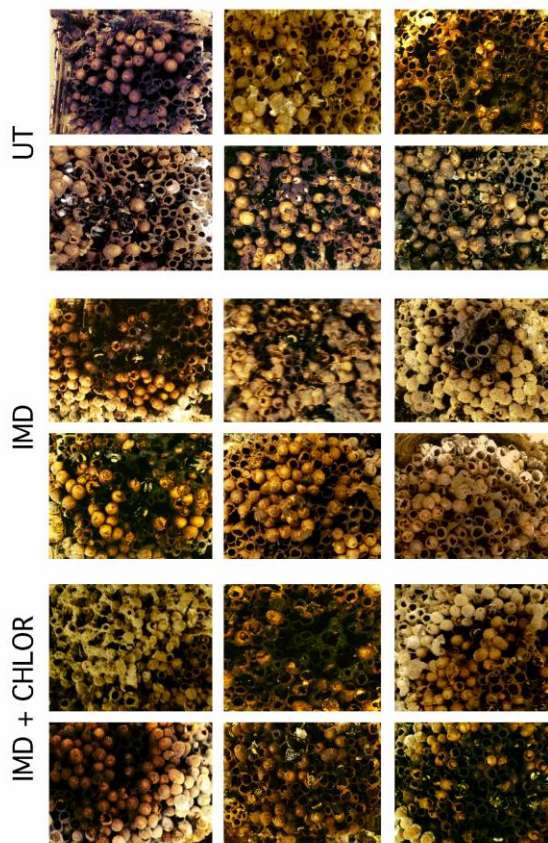

Supplement: Supplemental Data [file supp_fj.14-267179_Supplemental_Figure2.pdf]
