# Supplementary material for: Chronic exposure to neonicotinoids increases neuronal vulnerability to mitochondrial dysfunction in the bumblebee (Bombus terrestris)
Source: FASEB J. 2015 Jan 29;29(5):2112–9. doi: 10.1096/fj.14-267179 (PMC4415021; doi:10.1096/fj.14-267179)
Supplement: Supplemental Data [file supp_fj.14-267179_Supplemental_Table1.doc]

**SUPPLEMENTAL MATERIAL**

|  | **Crop Area** | |  | **Region** |
| --- | --- | --- | --- | --- |
|  | Kg (estimated) | Kg/ha |  | Kg/ha |
|  |  |  |  |  |
| **Arable Pesticide Usage Survey (2010)** | **East Fife** | | | |
| Pesticides | 150016 | 3.519 |  | 1.347 |
| Insecticides | 1448 | 0.034 |  | 0.013 |
| Neonicotinoids | 182 | 0.004 |  | 0.002 |
| Organophosphates | 545 | 0.013 |  | 0.005 |
|  |  |  |  |  |
|  | **Highlands and Islands** | | | |
| Pesticides | 6819 | 1.138 |  | 0.002 |
| Insecticides | 10 | 0.002 |  | 2.48e-6 |
| Neonicotinoids | 0 | 0 |  | 0 |
| Organophosphates | 0 | 0 |  | 0 |
|  |  |  |  |  |
| **Grassland Pesticide Usage Survey (2009)** | **East Fife** | | | |
| Pesticides | 2800 | 0.099 |  | 0.025 |
| Insecticides | 0 | 0 |  | 0 |
| Neonicotinoids | 0 | 0 |  | 0 |
| Organophosphates | 0 | 0 |  | 0 |
|  |  |  |  |  |
|  | **Highlands and Islands** | | | |
| Pesticides | 36288 | 0.014 |  | 0.009 |
| Insecticides | 0 | 0 |  | 0 |
| Neonicotinoids | 0 | 0 |  | 0 |
| Organophosphates | 0 | 0 |  | 0 |

**Table S1. Pesticide usage in location of field experiment.** Local pesticide load information is not available in the UK. To estimate likely environmental exposure to all pesticides, insecticides and in particular neonicotinoids and organophosphates, estimates of regional use (by SASA) were used to indicate likely local environmental levels. Estimated application rates were expressed as rates per hectare (ha) of crop area and rates per hectare of total regional area. These regional estimates were based on a limited number of samples. For arable, estimates the number of holdings and crop area surveyed were East Fife (27, 4556 ha) and grassland (13, 1063 ha) and Highlands & Islands arable (8, 259 ha) and grassland (77, 68949 ha). It should be noted that many estimates are based on less than 4% of the total area grown. The total area of the regions are 111,341 ha (East Fife) and 4,038,429 ha (Highlands & Islands). Arable crops include cereals, oilseed rape, potatoes, peas and field beans.

Fi**gure S1.** Quantification of IMD using a stable isotope dilution LC-MS/MS method. **A-B**. MS/MS spectra of IMD and d4-IMD. Data were acquired using a product ion scan with setting the same as described in Methods except that the product ion was scanned between 50-260 amu. Two dominant ions were observed in both IMD and d4-IMD and were monitored in a multiple reaction-monitoring mode. **C**. Calibration curve for IMD. **D.** IMD concentrations in brains of bees fed with IMD (see Materials and Methods for details) based on two transitions.

**Figure S2. Bumblebee nest condition.** Photographic images of the external face of each nest on the final day of the experiment. The images illustrate the visual condition of all 36 nests at the end of the field experiments. In the first experiment (left hand panels), a clear infestation with fungi and limited healthy brood was evident in the imidacloprid + Chlorpyrifos (IMD + CHLOR), but not in the untreated (UT) or chlorpyrifos (CHLOR) colonies. In the second experiment (right hand panels), there was a basal fungal contamination for all conditions, but this was far more extensive for imidacloprid treated (IMD and IMD + CHLOR) colonies. In addition, three imidacloprid treated colonies were invaded by wasps (Vespula vulgaris). In addition to the visual deterioration of the imidacloprid treated nests, we also identified an increased smell of decomposition and microbial infection in these colonies.​
